# Novel Substituted Heteroaromatic Piperazine and Piperidine Derivatives as Inhibitors of Human Enterovirus 71 and Coxsackievirus A16

**DOI:** 10.3390/molecules18055059

**Published:** 2013-04-29

**Authors:** Xian Zhang, Hongliang Wang, Yuhuan Li, Ruiyuan Cao, Wu Zhong, Zhibing Zheng, Gang Wang, Junhai Xiao, Song Li

**Affiliations:** 1 Key Laboratory of Structure-Based Drug Design and Discovery of the Ministry of Education, School of Pharmaceutical Engineering, Shenyang Pharmaceutical University, Shenyang 110016, Liangning, China; E-Mails: zhangxian85@163.com (X.Z.); wg0115@sina.com (G.W.); 2 National Engineering Research Center for the Strategic Drugs, Beijing Institute of Pharmacology and Toxicology, Beijing 100850, China; E-Mails: whl83319@126.com (H.W.); 21cc@163.com (R.C.); zhongwu@bmi.ac.cn (W.Z.); zzbcaptain@aliyun.com (Z.Z.); 3 Institute of Medicinal Biotechnology, Chinese Academy of Medical Sciences and Peking Union Medical College, Beijing 100050, China; E-Mail: yuhuanlibj@126.com

**Keywords:** virtual screening, piperazine derivative, piperidine derivatives, anti-EV71 activity, anti-CVA16 activity

## Abstract

A series of substituted heteroaromatic piperazine and piperidine derivatives were found through virtual screening based on the structure of human enterovirus 71 capsid protein VP1. The preliminary biological evaluation revealed that compounds **8e** and **9e** have potent activity against EV71 and Coxsackievirus A16 with low cytotoxicity.

## 1. Introduction

Enterovirus 71 (EV71) and Coxsackievirus A16 (CVA16) belong to the genus Enterovirus of family Picornaviridae [1]. Both viruses are common causes of hand, foot, and mouth disease (HFMD), which mostly affects young children [2]. The clinical manifestations associated with HFMD caused by both viruses include fever, sore throat, diarrhea, and papulovesicular rash on the hands, feet, and oropharyngeal mucosa. Moreover, EV71 infections can damage the central nervous system, leading to viral meningitis, encephalitis, or severe myocarditis with or without fatal pulmonary edema [3,4,5]. Many epidemic outbreaks have been reported in Southeast and East Asia because of the high of morbidity and mortality rates associated with such infections. Between January 2009 and May 2011, the EV71 epidemic in China has led to 1,000 deaths [6]. Although few inhibitors and vaccines have been reported, such as compound **1,** shown in Supplementary Figure S1 [7,8,9,10], no antiviral agent has been approved by the US Food and Drug Administration for treating HFMD caused by enteroviral infections. Clinical treatments are directed only toward relieving the most prominent symptoms of each clinical syndrome. Therefore, we need to develop novel EV71 and CVA16 inhibitors.

In recent years, there are some organic molecules were identified for HFMD therapy [11], and EV71-related anti-virus activity had been identified by screening traditional Chinese medicines [12,13]. EV71 and CVA16, which are non-enveloped viruses, comprise a positive single-stranded RNA genome packed within an outer capsid composed of four proteins (VP1–VP4) [1]. Mature EV71, like other enteroviruses, has a hydrophobic pocket on VP1 that penetrates from the surface deep into the interior of the VP1 β-barrel, which underlies a canyon-like surface depression [14] and harbors a natural lipid (possibly sphingosine) called the “pocket factor” [15]. Releasing the pocket factor contributes to activation because it is required for initiating the uncoating and liberation of the RNA genome; thus, potential antivirals simulate natural lipids and inhibit uncoating [16], which provides a theoretical basis for virtual screening. 

Our laboratory built an in-house chemical database of nearly 10,000 compounds. Based on the crystal structure of the EV71 capsid protein [Protein Data Bank (PDB) ID: 3VBH], we screened our database using DOCK 6.0 and found novel N-containing heterocyclic piperazines and piperidines as EV71 inhibitors and CVA16 inhibitors. Our findings provide a basis for anti-EV71 and anti-CVA16 drug development. 

## 2. Results and Discussion

### 2.1. Computer Screening

Identifying a suitable site on the VP1 protein for binding small organic ligands is critical for successfully implementing the computer screening strategy for developing effective anti-EV71 inhibitors. The binding site is a hydrophobic pocket that penetrates from the surface deep into the interior of the VP1 β-barrel and harbors a natural lipid (possibly sphingosine) called the “pocket factor” by Wang [16]. The closure of the VP1 pocket after the pocket factor was expelled initiated EV71 uncoating with pocket collapse; which switches mature EV71 into expanded particles and transforms into a conformation with a high-receptor affinity. Therefore; the release of the pocket factor is required for initiating viral uncoating; and the discovery of potential anti-EV71 inhibitors focuses on compounds that are structurally similar to natural lipids to occupy the hydrophobic pocket; the binding site. Some amino acids are important in EV71 VP1 and the natural lipid complex crystal structure (PDB ID: 3VBH). The hydroxyl group of natural lipids was 2.88 Å away from the N atom of Ile113. It indicates the formation of H-bond. Morever the natural lipid and residues Ile111; Asp112; Thr114; Val192; Met230; and Phe233 interact through molecular electrostatic potential; and Van der Waals force. 

**Figure 1:**
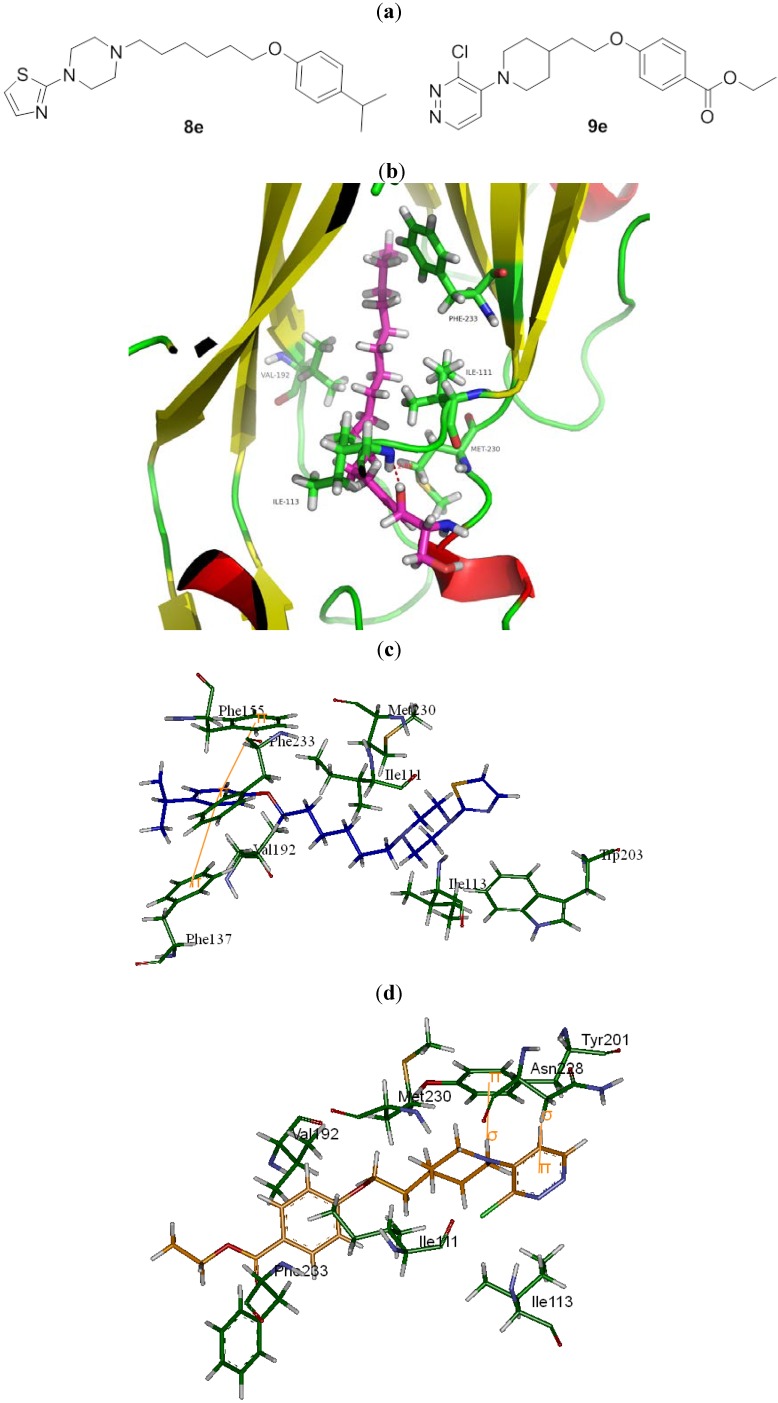
(**a**) Structures of compounds **8e**, and **9e**. (**b**) The natural lipid (pink) was docked in the hydrophobic pocket on the VP1 of EV71. The hydroxy group of the natural lipid was 2.88 Å from the N of Ile113 (green), which suggests a potential H-bond (red). (**c**) The structure of **8e** (blue) docked in the pocket with important interactions shown by orange lines. (**d**) The structure of **9e** (orange) docked in the pocket with important interactions shown by orange lines.

The identification of a VP1 capsid-binding pocket facilitated the computational screening of 10,000 organic compounds using our in-house chemical database for potential ligands for this pocket. This screening selected 60 compounds for cell culture cytopathic effect (CPE) assays. Most of the 60 compounds were known as HRV inhibitors before. It is not surprising since the homology of EV71 VP1 coat protein (Sequence ID: Q91PB1) with human rhinovirus (HRV) 2 coat protein (PDB ID: 1FPN) and HRV16 coat protein (PDB ID: 1AYM) from UniProtKB were up to 40% and 38%, respectively. Among the 60 cmpounds identified, **8e** is novel and **9e** is known to inhibit HRV and their docking models in the hydrophobic pocket are shown in Figure 1 as compared to compound **1** developed by Shih *et al.* [10] shown in Supplementary Figure S2, Figure S3. Ile111, Val192, Met230, and Phe233 formed hydrophobic interactions with different moieties of compounds **1**, **8e**, and **9e**. The carbonyl group of imidazolidinone in compound **1** was 3.49 Å away from the N atom of Ile113, indicating formation of H-bond. It was also observed that the formation of π-π effects between the compound **1** and residues Phe155 and Trp203 existed. Besides, Phe137, Phe155 and **8e** interacted through π-π effect, whereas Tyr201 and Asn228 performed σ-π effect with **9e**. It seems that those compounds have more potential for EV71 VP1 inhibition. Fifteen of these compounds displayed significant inhibitory effect (median inhibitory concentration [IC_50_]: 1 μM to 100 μM), with the structure of the 15 organic inhibitors shown in Table 1. This finding also indicates further structural optimization directions for our research.

**Table 1:** Anti-EV71 activity, anti-CVA16 activity, and cytotoxicity of compounds **8a**–**f** and compounds **9a**–**h**. 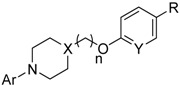

Cpd	Ar	X	Y	n	R	TC_50_ (μM)	TC_0_ (μM)	EV71		CVA16	
								IC_50_ (μM)	SI	IC_50_ (μM)	SI
1	-	-	-	-	-	78 ± 5.3	7.7 ± 0.5	0.6 ± 0.0	137	>7.7	-
9a	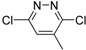	N	C	2	C_4_H_9_	19.2 ± 0.7	7.7 ± 0.3	1.2 ± 0.0	16.1	1.4 ± 0.1	13.8
9b	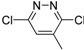	N	C	2	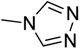	189 ± 17.3	29.7 ± 2.2	21.6 ± 1.4	8.7	>29.7	-
9c	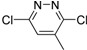	N	C	2	OC_2_H_5_	62.9 ± 2.4	31.5 ± 2.1	>31.5	_	10.8 ± 0.3	5.8
9d	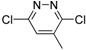	C	C	2	CO_2_CH_3_	193 ± 19.3	30.5 ± 2.0	>31.5	_	22.1 ± 1.1	8.7
9e	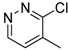	C	C	2	CO_2_C_2_H_5_	>513	>513	1.0 ± 0.2	513	12.7 ± 0.4	>40.3
9f	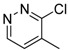	C	N	2	CO_2_C_2_H_5_	>512	128 ± 9.5	25.4 ± 1.7	>20.2	16.0 ± 0.9	>32.0
9g	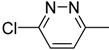	N	C	3	CO_2_CH_3_	256 ± 22.7	128 ± 3.8	16.0 ± 0.6	16.0	50.8 ± 4.1	5.0
9h	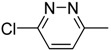	N	C	3	CO_2_C_2_H_5_	359 ± 21.5	31 ± 4.3	4.9 ± 0.2	73.7	4.9 ± 0.2	73.7
8a	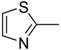	N	C	5	OCH_3_	277 ± 2.2	138 ± 9.6	100 ± 9.0	2.8	69.2 ± 5.5	4.0
8b	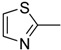	N	C	5	OC_2_H_5_	66.6 ± 1.0	33.3 ± 3.9	21 ± 1.2	3.2	5.3 ± 0.3	12.7
8c	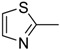	N	C	6	OC_2_H_5_	54.1 ± 8.1	6.8 ± 0.1	4.3 ± 0.1	12.7	6.8 ± 0.4	8.0
8d	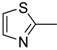	N	C	6	C_2_H_5_	56.0 ± 3.3	28.0±2.5	4.4 ± 0.3	12.7	14.0 ± 0.5	4.0
8e	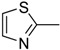	N	C	6	CH(CH_3_)_2_	43.1 ± 4.7	6.8 ± 0.8	4.3 ± 0.0	10.1	1.2 ± 0.5	34.8
8f	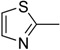	N	C	6	C(CH_3_)_3_	15.6 ± 0.3	7.8 ± 0.4	3.9 ± 0.1	4.0	7.8 ± 0.6	2.0

### 2.2. Chemistry

**Scheme 1:**
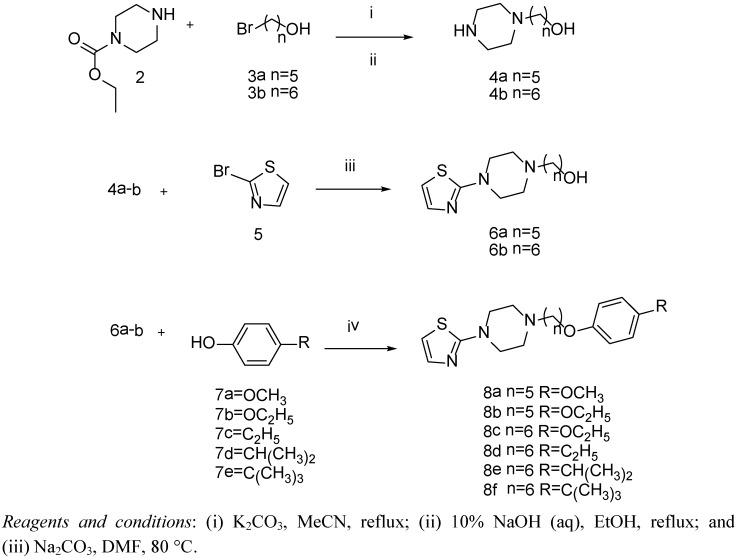
Synthesis of compounds **8a**–**f**.

Compounds **9a**–**h** (Table 1) were previously reported as HRV inhibitors [17,18]. Because compounds **8a**–**f** have never been reported, we describe their synthesis in this paper (Scheme 1). To prepare piperazine-1-yl-alkyl alcohol **4a**–**b**, ethyl piperazine-1-carboxylate **2** was coupled with compounds **3a**–**b** in the presence of K_2_CO_3_ in acetonitrile and exposed the ethoxycarbonyl group in the presence of aq NaOH in ethanol. The 2-bromo group of 2-bromothiazole (**5**) was substituted with compounds **4a**–**b** in the presence of Na_2_CO_3_ in dimethylformamide (DMF) to yield compounds **6a**–**b**. Finally, compounds **8a**–**f** were synthesized through the Mitsunobu reaction by coupling compounds **6a**–**b** and 4-substituted phenols **7a**–**e**.

### 2.3. Biology

The anti-EV71 activity, anti-CVA16 activity, and cytotoxicity of the compounds on the EV71 test strain (Shenzhen 98, shzh01-8) and CVA16 (laboratory viral culture) were evaluated by using cell culture cytopathic effect assays. The results are listed in Table 1.

#### 2.3.1. Results for Anti-EV71 Activity

In the series of imidazolidinone compounds reported by Shia [10], the length of the alkyl linker does not contribute significantly to antiviral activity. However, we found such length to be important, as evidenced by our compounds **8b** and **8c**. The *p*-monosubstituted phenyl ring crucially affects antiviral activity [10]. Similarly, the steric requirement of 4-electron donating substitution may have affected the antiviral activity against EV71 via **8d**–**f**. Moreover, the larger the alkoxy group, the higher the activity, such as with compounds **8a**–**b** and **9g**–**h**. Compared with that of its corresponding pyridine isomer **9f**, the antiviral activity of the benzene ring of **9e** was increased 25-fold. This effect might be due to the strict steric requirement on the phenyl ring or to the increased in basicity of the pyridine nitrogen. Although chloropyridine derivatives **9a**–**h** have been known as potent human rhinovirus capsid-binding inhibitors [17,18], it hasn’t been reported that the series of compounds manifests anti-EV71 activity. We first discovered compounds **9a**–**h** showed a wide range of anti-EV71 activity. **9a**–**f** were the derivatives of pirodavir that was known as the anti-HRV capsid-binding inbititor [19] (Supplementary Figure S1, IC_50_ = 1.2 ± 0.2 μM, TC_50_ = 31 ± 2.2 μM, SI = 25 [20]). However, compound **9e** exhibited high antiviral potency and an excellent selectivity index (IC_50_ = 1.0 μM, TC_50_ > 512.97 μM, SI = 512.8). 

#### 2.3.2. Results for anti-CVA16 Activity

The evaluation of the series of compounds **8b**–**c** revealed that the length of the alkyl linker was not sensitive to anti-CVA16 activity. **9b** exhibited moderate anti-EV71 activity, but it was inactive at the highest concentration (29.74 μM, TC_0_ = 29.74 μM). The compounds with the alkoxy, ester, or alkyl group at position 4 of the phenoxyl ring (compounds **8a**–**f** and **9g**–**h**) revealed that the suitable steric volume, neither too large nor too small, may be significant for anti-CVA16. Compound **9e** was similar to its corresponding pyridine isomer **9f** in terms of anti-coxsackie activity. These effects might be due to distinct differences in the hydrophobicity of the compounds. It was the first time that their anti-CVA16 activity was reported.

## 3. Experimental

### 3.1. General

Melting points (mp; °C, uncorrected) were determined in open glass capillaries using a YRT-3 electrothermal melting point apparatus. The ^1^H-NMR and ^13^C-NMR were recorded on a Bruker ARX 400 MHz spectrometer (Karlsruhe, Germany). Chemical shifts are expressed in δ (ppm) with reference to tetramethylsilane. The mass spectra were processed through a Waters Xevo G2 QTof electrospray ionization (ESI) spectrometer (Denver, CO, USA). All solvents and reagents were purchased commercially and used without further purification.

### 3.2. Computer Screening

The high-resolution X-ray structure of EV71 VP1 with the capsid-binding pocket was used as a receptor for ligand docking. DOCK 6.0 is an automatic computerized method used to screen small molecule databases for ligands that bind to a given receptor [21]. DOCK 6.0 defined the EV71 VP1 binding site with a set of overlapping spheres, the centers of which became the potential locations for ligand atoms. The binding of an organic ligand to the VP1 hydrophobic pocket was evaluated based on shape complementarity and simplified interaction energy (force field energy). Our in-house database was chosen as the small-molecule database because it included approximately 10,000 available small organic compounds. The molecular structures were generated using the CONCORD heuristic algorithm (developed by R. Pearlman at the University of Texas). The parameters used for docking were set to the defaults. For example, the maximum orientation was 1500, and the bump_max was 3. Following four rounds of inflexible and flexible virtual screens, 50 molecules with the best shape complementarity scores and 50 with the best force field scores were selected from the DOCK 6.0 screening. The resulting 60 compounds were then chosen for the cell culture CPE assay. Of these 60 compounds, 37 were from the shape list, 23 were from the force field list, and 9 were on both lists.

### 3.3. Chemical Synthesis

#### 3.3.1. Synthesis of Compounds **4a**,**b**, and **6a**,**b**

*5-(4-(Thiazol-2-yl)piperazin-1-yl)pentan-1-ol* (**6a**). Ethyl piperazine-1-carboxylate **2** (25.5 g, 161.39 mmol), 5-bromopentan-1-ol (22.43 g, 161.39 mmol), and K_2_CO_3_ (55.68 g, 403.48 mmol) were refluxed overnight in 200 mL of acetonitrile. Upon cooling, the reaction mixture was filtered to remove insoluble solids, and the filtrate was evaporated *in vacuo* to remove the acetonitrile to produce an oily residue. The crude product was purified via column chromatography (CH_2_Cl_2_/MeOH/Et_3_N 100:1:0.5) to afford intermediate ethyl 4-(5-hydroxypentyl)piperazine-1-carboxylate as a yellow oil (20.48 g, 52.0%). Ethyl 4-(5-hydroxypentyl)piperazine-1-carboxylate (15.9 g, 65.09 mmol) and 10% aq NaOH (150 mL) were then refluxed overnight in 150 mL of EtOH. The solvent was removed under reduced pressure, and the crude residue was diluted with 200 mL of brine and extracted with dichlormethane (5 × 200 mL). The organic layer was dried over MgSO_4_, filtered, and concentrated. The resulting yellow oil **4a** (9.69 g, 86.5%) was used for the next step without further purification. **4a** (8.6 g, 50 mmol) in DMF (10 mL) was added to a stirred suspension of 2-bromothiazole (12.30 g, 75 mmol) and Na_2_CO_3_ (5.3 g, 50 mmol) in DMF (40 mL) at 0 °C in a three-necked flask. The resulting mixture was stirred overnight at 80 °C and removed *in vacuo* to yield a yellow solid. The residue was washed with ether (3 × 200 mL) and recrystallized with petroleum ether to yield a white solid **6a** (5.95 g, 46.5%).

*6-(4-(thiazol-2-yl)piperazin-1-yl)hexan-1-ol* (**6b**). Compound **6b** was obtained as a white solid (6.84 g, 21.4%) from 6-bromohexane-1-ol according to the same procedure.

#### 3.3.2. Preparation of Compounds **8a–f**

*2-(4-(5-(4-Methoxyphenoxy)pentyl)piperazin-1-yl)thiazole* (**8a**). Diethyl azodicarboxylate (0.35 g, 2 mmol) was added to a solution of **6a** (0.51 g, 2 mmol), 4-methoxyphenol (0.22 g, 2 mmol), and triphenylphosphine (0.52 g, 2 mmol) in dry tetrahydrofuran (10 mL) at 0 °C in a three-necked bottle. The resulting mixture was stirred overnight at ambient temperature. The solvent was then removed under reduced pressure to yield a crude product, which was then purified via column chromatography (petroleum ether/acetone 15:1) to provide a white solid of **8a** (0.15 g, 20.7%): mp = 83 °C to 85 °C; ^1^H-NMR (CDCl_3_): δ7.20 (d, 1H, *J* = 3.6 Hz), 6.83 (s, 4H), 6.58 (d, 1H, *J* = 3.2 Hz), 3.92 (t, 2H, *J* = 6.4 Hz), 3.77 (s, 3H), 3.52 (br, 3H), 2.57 (br, 3H), 2.42 (br, 1H), 1.80 (m, 2H), and 1.50–1.60 (m, 4H); ^1^^3^C-NMR (dimethyl sulfoxide [DMSO]): δ171.37, 153.20, 152.67, 139.49, 115.25, 114.57, 108.08, 67.75, 57.72, 55.34, 51.92, 48.30, 28.72, 26.01, and 23.53; ESI-HRMS: *m/z* [M+H]^+^ calculated for C_19_H_27_N_3_O_2_S: 362.1902; found: 362.1902.

*2-(4-(5-(4-Ethoxyphenoxy)pentyl)piperazin-1-yl)thiazole* (**8b**). Compound **8b** was obtained as a white solid (0.25 g, 33.3%) from compound **6a** according to the same procedure: mp = 79 °C to 81 °C; ^1^H-NMR (CDCl_3_): δ7.20(d, 1H, *J* = 3.6 Hz), 6.82 (s, 4H) , 6.57 (d, 1H, *J* = 3.2 Hz), 3.90–4.00 (m, 4H), 3.52 (br, 4H), 2.57 (br, 4H), 2.43 (br, 2H), 1.79 (m, 2H), 1.49–1.60 (m, 4H), and 1.39 (t, 3H); ^1^^3^C-NMR (DMSO): δ171.36, 152.60, 152.43, 139.46, 115.22, 115.15, 108.05, 67.72, 63.26, 57.71, 51.91, 48.29, 28.71, 26.00, 23.52, and 14.78; ESI-HRMS: *m/z* [M+H]^+^ calculated for C_20_H_29_N_3_O_2_S: 376.2059; found: 376.2061. 

*2-(4-(6-(4-Ethoxyphenoxy)hexyl)piperazin-1-yl)thiazole hydrochloride* (**8c**). Diethyl azodicarboxylate (0.35 g, 2 mmol) was added to a solution of **6b** (0.54g, 2 mmol), 4-ethoxyphenol (0.28g, 2 mmol), and triphenylphosphine (0.52 g, 2 mmol) in dry tetrahydrofuran (10 mL) at 0 °C in a three-necked bottle. The resulting mixture was stirred overnight at ambient temperature. The solvent was then removed under reduced pressure to yield a crude product, which was then purified via column chromatography (petroleum ether/acetone 15:1) to provide a white solid of **8c** (0.32 g). The white solid was added to acetone (10 mL) to form a transparent solution. A saturated solution of hydrogen chloride in ether was added dropwise to this solution until the pH was adjusted to about 5. The resultant precipitate was filtered and dried to yield a white solid **8c** (0.38 g, yield 41.1%): mp = 222 °C to 224 °C; ^1^H-NMR (DMSO): δ10.99 (s, 1H), 7.26 (d, 1H, *J* = 3.6 Hz), 7.00 (d, 1H, *J* = 4.0), 6.83 (s, 4H), 3.88–3.95 (m, 6H), 3.49–3.57 (m, 4H), 3.09–3.14 (m, 4H), 1.68–1.73 (m, 4H), and 1.29–1.44 (m, 3H); ^13^C-NMR (DMSO): δ170.54, 153.05, 152.96, 135.37, 115.71, 115.67, 110.35, 68.12, 63.77, 55.76, 49.87, 46.13, 29.02, 26.32, 25.59, 23.36, and 15.29; ESI-HRMS: *m/z* [M+H]^+^ calculated for C_21_H_33_Cl_2_N_3_O_2_S: 390.2213; found: 390.2215.

*2-(4-(6-(4-Ethylphenoxy)hexyl)piperazin-1-yl)thiazole hydrochloride* (**8d**). 2-(4-(6-(4-Ethylphenoxy)-hexyl)piperazin-1-yl)thiazole hydrochloride was obtained as a white solid (0.12 g, yield 13.4%) from compound **6b** according to the same procedure: mp = 240 °C to 242 °C; ^1^H-NMR (DMSO): δ 11.32 (s, 1H), 7.27 (d, 1H, *J* = 3.6 Hz), 7.06 (d, 2H, *J* = 8.4), 7.01 (d, 1H, J = 3.6), 6.79 (d, 2H, J = 8.4), 3.86–4.03 (m, 4H), 3.52–3.60 (m, 4H), 3.04–3.09 (m, 4H), 1.67–1.72 (m, 4H), 1.32–1.40 (m, 4H), and 1.09 (t, 3H, J = 7.6 Hz); ^1^^3^C-NMR (DMSO): 170.26, 156.70, 137.08, 135.57, 128.65, 114.25, 109.86, 67.17, 55.29, 49.55, 45.37, 28.48, 27.31, 25.81, 25.10, 22.93, and 16.03; ESI-HRMS: *m/z* [M+H]^+^ calculated for C_21_H_33_Cl_2_N_3_OS: 374.2266; found: 374.2275.

*2-(4-(6-(4-Isopropylphenoxy)hexyl)piperazin-1-yl)thiazole hydrochloride* (**8e**). 2-(4-(6-(4-isopropylphenoxy)hexyl)piperazin-1-yl)thiazole hydrochloride was obtained as a white solid (0.33 g, yield 35.8%) from compound **6b** according to the same procedure.: mp = 135 °C to 137 °C; ^1^H-NMR (DMSO): δ10.93 (s, 1H), 7.23 (d, 1H, *J* = 3.6 Hz), 7.13 (d, 2H, *J* = 8.4 Hz), 6.98 (d, 1H, *J* = 4.0), 6.83 (d, 2H, *J* = 8.4), 3.91–4.00 (m, 4H), 3.45–3.57 (m, 4H), 3.08–3.13 (m, 4H), 2.82 (m, 3H), 1.68–1.74 (m, 4H), 1.35–1.47 (m, 4H), and 1.16 (d, 6H, *J* = 6.8 Hz); ^1^^3^C-NMR (DMSO): 170.44, 156.75, 140.25, 139.55, 127.14, 114.19, 109.84, 67.16, 55.32, 49.75, 45.10, 32.59, 28.47, 25.80, 25.12, 24.20, and 22.98; ESI-HRMS: *m/z* [M+H]^+^ calculated for C_22_H_35_Cl_2_N_3_OS: 388.2423; found: 388.2429. 

*2-(4-(6-(4-tert-Butylphenoxy)hexyl)piperazin-1-yl)thiazole* (**8f**). Compound **8f** was obtained as a white solid (0.33 g, 63.0%) from compound **6b** according to the same procedure: mp = 30 °C to 32 °C; ^1^H-NMR (CDCl_3_): δ7.29 (t, 2H, *J* = 2.0 Hz), 7.20 (d, 1H, *J* = 3.6 Hz), 6.83 (d, 2H, *J* = 8.8 Hz), 6.57 (d, 1H, *J* = 3.6 Hz), 3.94 (t, 2H, *J* = 6.4 Hz), 3.52 (br, 4H), 2.57 (br, 4H), 2.41 (br, 2H), 1.78 (m, 2H), 1.37–1.58 (m, 6H), and 1.30 (s, 9H); ^1^^3^C-NMR (DMSO): 171.37, 156.42, 142.44, 139.46, 126.02, 113.83, 108.04, 67.23, 57.73, 51.92, 48.28, 33.74, 31.38, 26.69, 26.21, and 25.51; ESI-HRMS: *m/z* [M+H]^+^ calculated for C_23_H_35_N_3_OS: 402.2579; found: 402.2563.

### 3.4. Biological Evaluation

#### 3.4.1. Neutralization Test [22]

This assay measured the ability of the test compounds to inhibit the CPE induced by EV71 and CVA16 on Vero cells. The 96-well tissue culture plates were seeded with 20,000 Vero cells/mL. The plates were incubated for 24 h at 37 °C. The virus (100 TCID_50_) mixed with different concentrations of the test compounds was added to the cells, which were incubated at 37 °C for 2 h. After adsorption, the infected cells were overlaid with 50 μL of Dulbecco’s modified Eagle’s medium with 5% fetal bovine serum, and 2% DMSO. The concentration of the test compound required to reduce the virus-induced CPE to 4+ relative to the virus control was expressed as IC_50_ according to the Reed–Muench method. All assays were performed in triplicate.

#### 3.4.2. Cytotoxicity Assay [23]

The Vero cells treated with the test compounds were incubated at 37 °C for 96 h. After incubation, the cells were harvested and counted. The concentration of a test compound required to reduce cell viability to 50% of the tested control culture was expressed as TC_50_.

## 4. Conclusions

A computer-based database screening strategy was employed to identify a novel series of aromatic heterocyclic substituted piperazine and piperidine derivatives as ligands for the EV71 VP1 hydrophobic pocket that exhibits antiviral activity against EV71 and CVA16. Further mechanistic studies on this new class of anti-EV71 inhibitors are in progress. Our study on the structure–activity relationships of anti-EV71 and anti-CVA16 indicated that the space volume of the 4-electron donating group substituent at the phenoxyl ring largely influenced the *in vitro* anti-EV71 activity of the new class of potent inhibitors, and the appropriate steric requirement on the *p*-monosubstituted phenyl ring may exhibit activity against CVA16. Interestingly, the anti-EV71 activity of this series of compounds was reduced when the phenoxyl ring was substituted for the corresponding pridine isomer. Moreover, the length of the alkyl linker influenced the anti-EV71 activity. However, both factors did not affect the anti-CVA16 activity. The piperidine derivative **9e** exhibited the most potent antiviral activity against EV71 (IC_50_ = 1 μM), with no apparent cytotoxicity against Vero cells (TC_50_ > 512.97 μM), compared with compound **1**(IC_50_ = 1 μM, TC_50_ = 77.91 μM). Compound **8e** exhibited excellent activity against CVA16 (IC_50_ = 1.24 μM) and moderate activity against EV71 (IC_50_ = 4.28 μM), with weak cytotoxicity on Vero cell lines (TC_50_ = 43.08 μM). Therefore, these compounds are very promising candidates for further optimization towards a drug for HFMD treatment.

## Supplementary Materials

Supplementary materials can be accessed at: http://www.mdpi.com/1420-3049/18/5/5059/s1.
