# Supplementary material for: Novel Substituted Heteroaromatic Piperazine and Piperidine Derivatives as Inhibitors of Human Enterovirus 71 and Coxsackievirus A16
Source: Molecules. 2013 Apr 29;18(5):5059–71. doi: 10.3390/molecules18055059 (PMC6269887; doi:10.3390/molecules18055059)

Supplementary Materials

The ^{1}H-NMR spectra were measured using a multinuclear Fourier transform (FT)-NMR spectrometer ARX400 (Bruker); Chemical shift are referenced to internal SiMe4.

Figure S1. The ^{1}H-NMR spectrum of compound **8a** in $CDCl_3$.

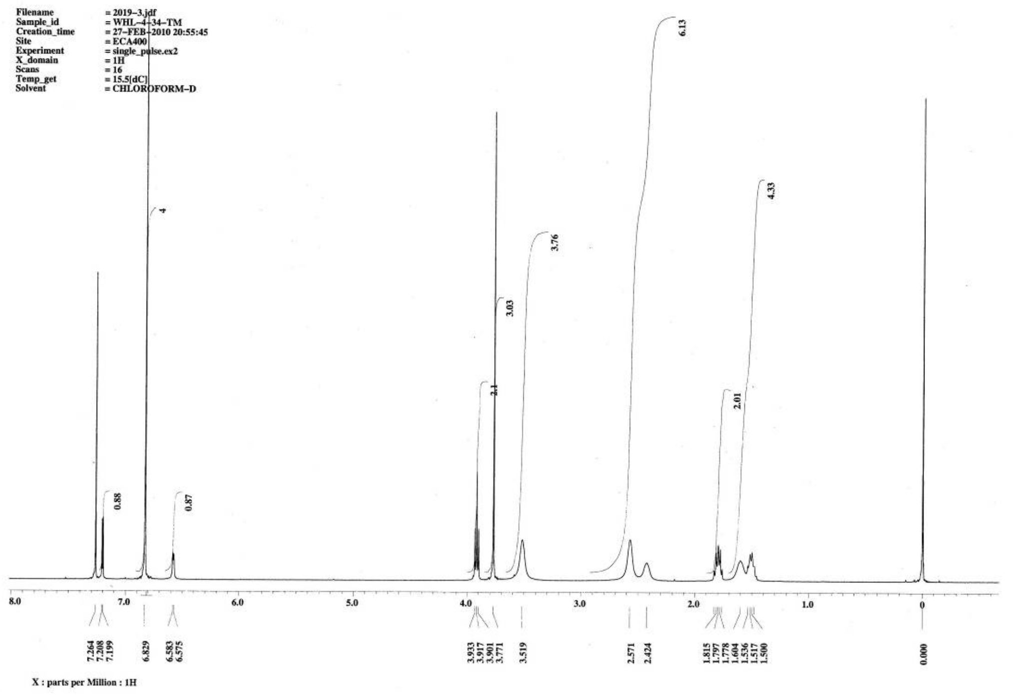

Figure S2. The ^{13}C-NMR spectrum of compound **8a** in DMSO.

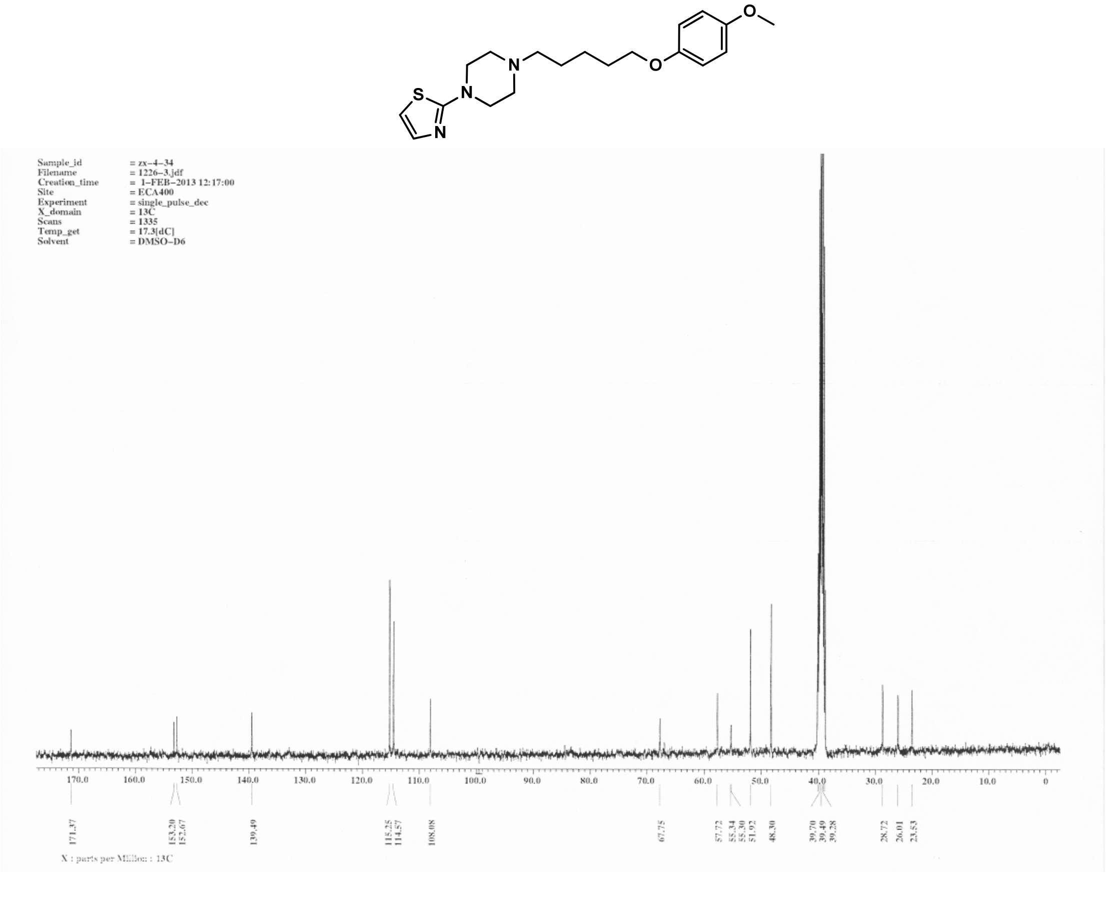

Figure S3. The ESI-HRMS of compound **8a**.

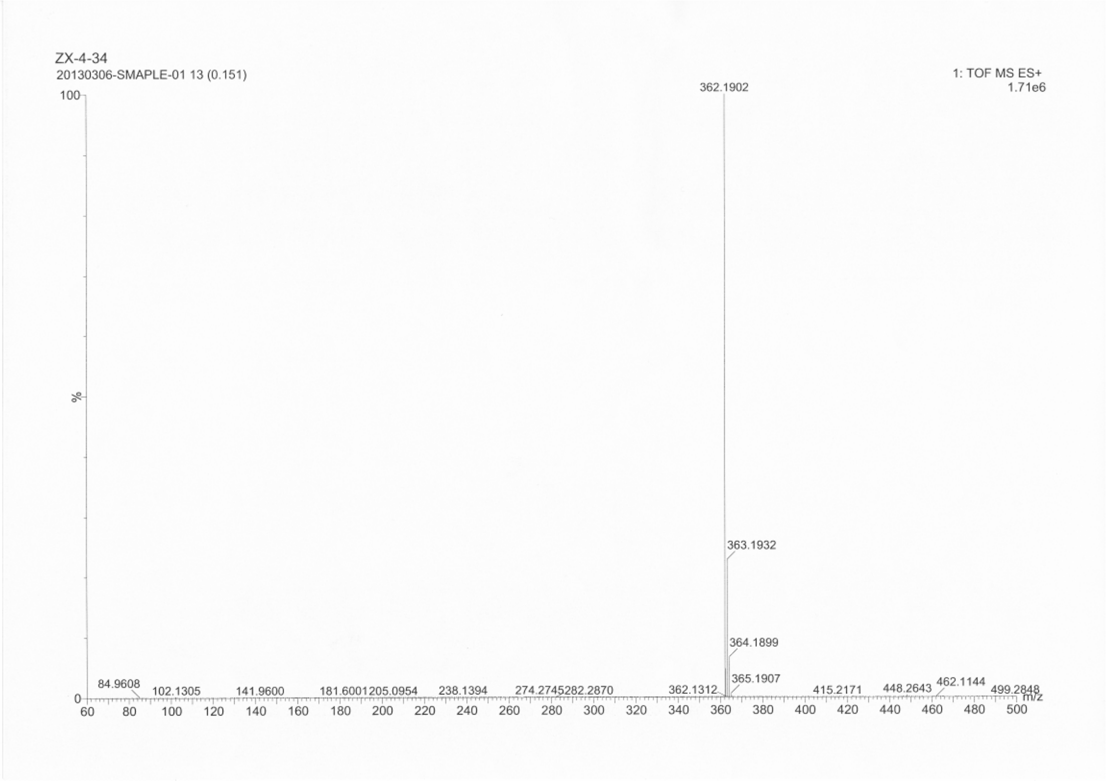

Figure S4. The 1H-NMR spectrum of compound **8b** in $CDCl_3$.

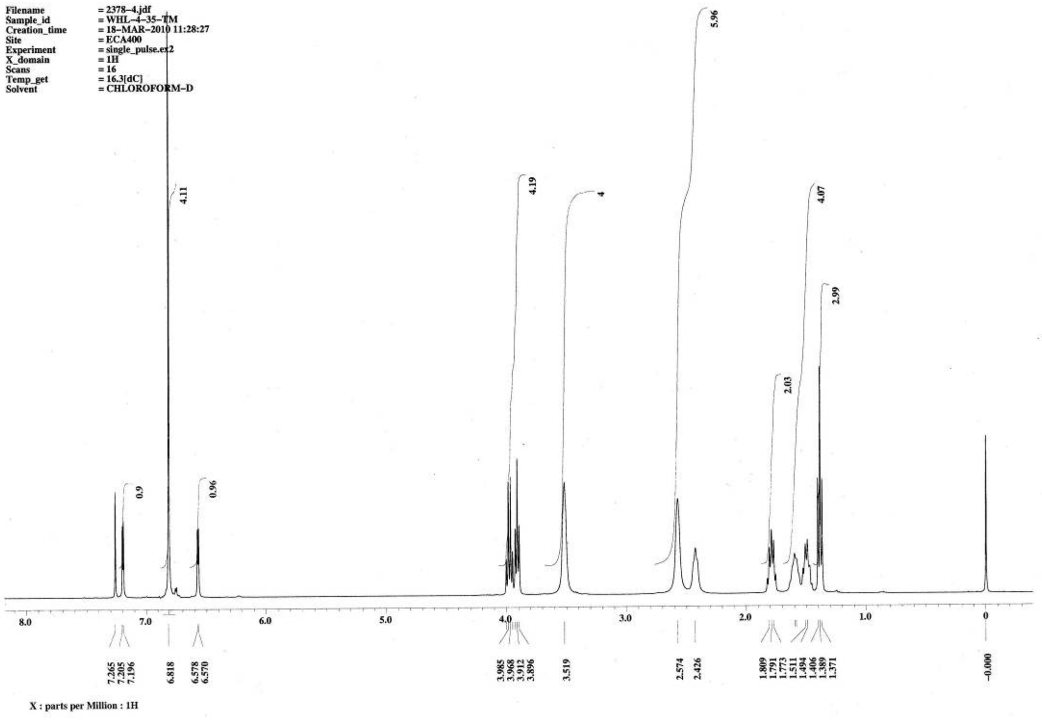

Figure S5. The ^{13}C-NMR spectrum of compound **8b** in DMSO.

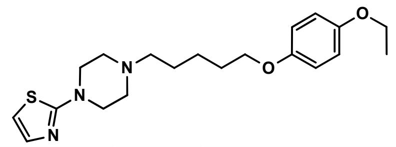

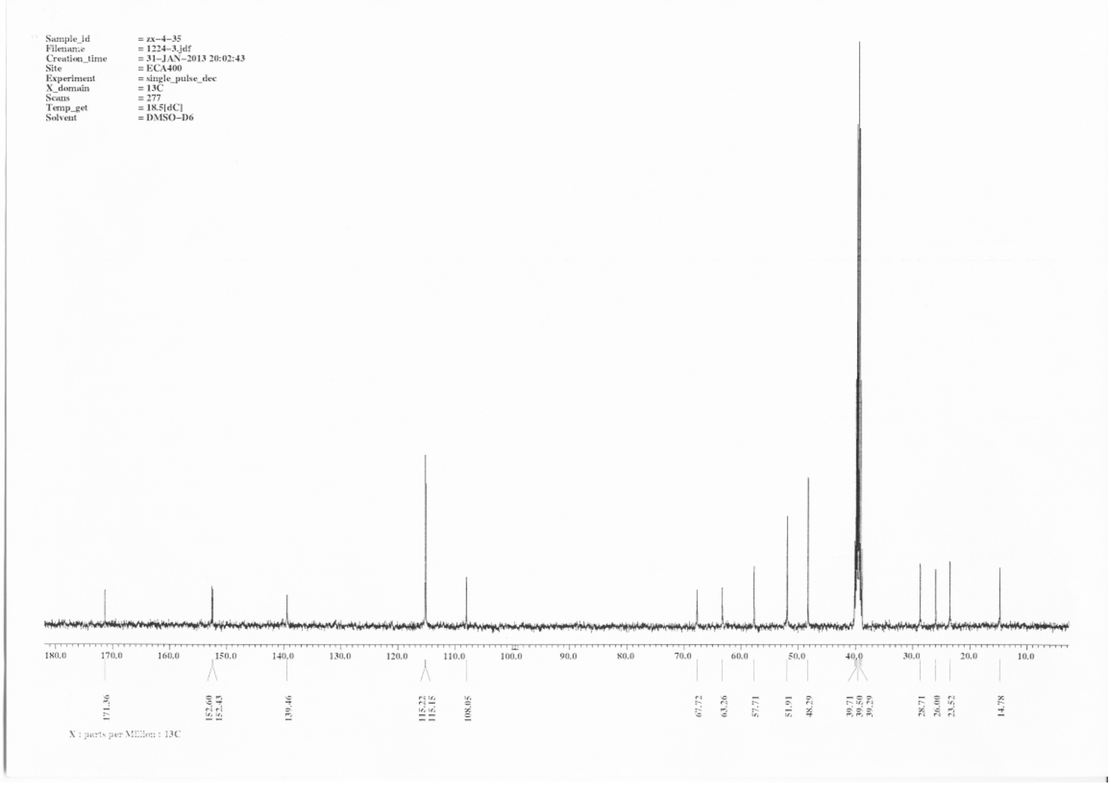

Figure S6. The ESI-HRMS of compound **8b**.

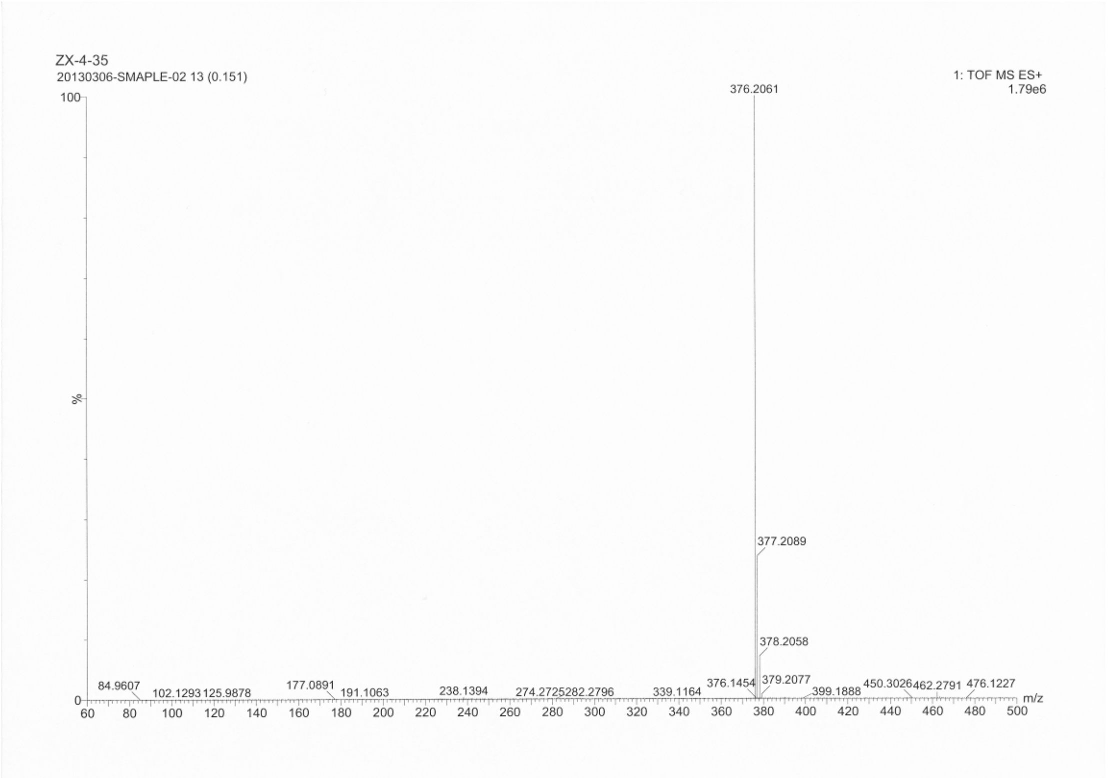

Figure S7. The ^{1}H-NMR spectrum of compound **8c** in DMSO.

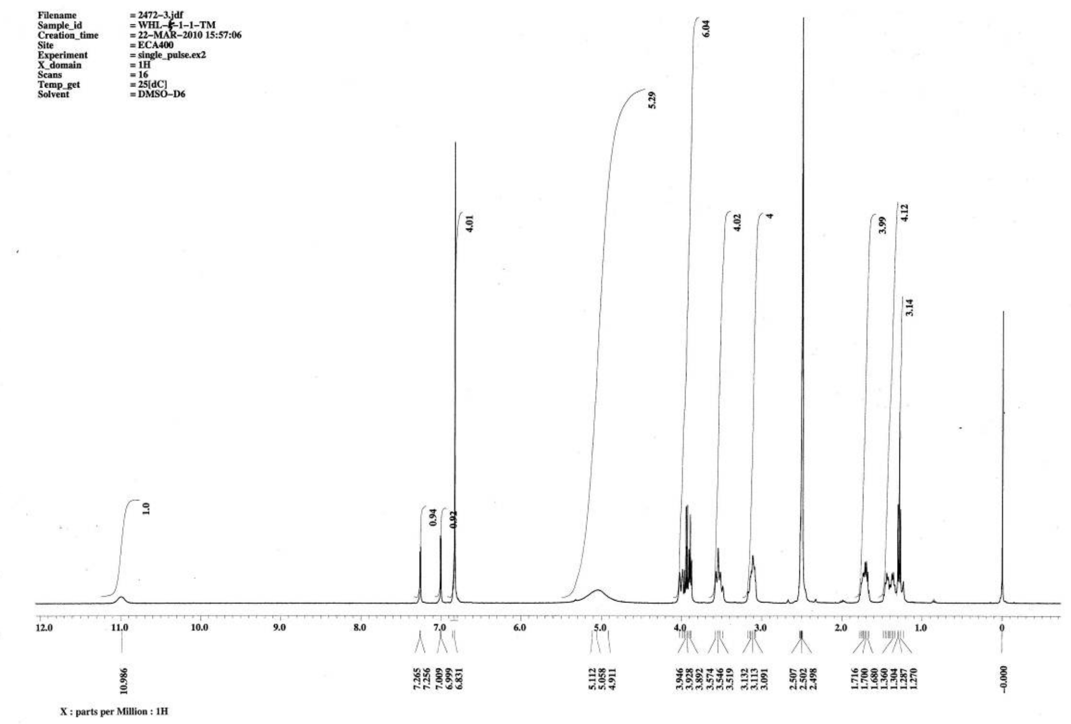

Figure S8. The ^{13}C-NMR spectrum of compound **8c** in DMSO.

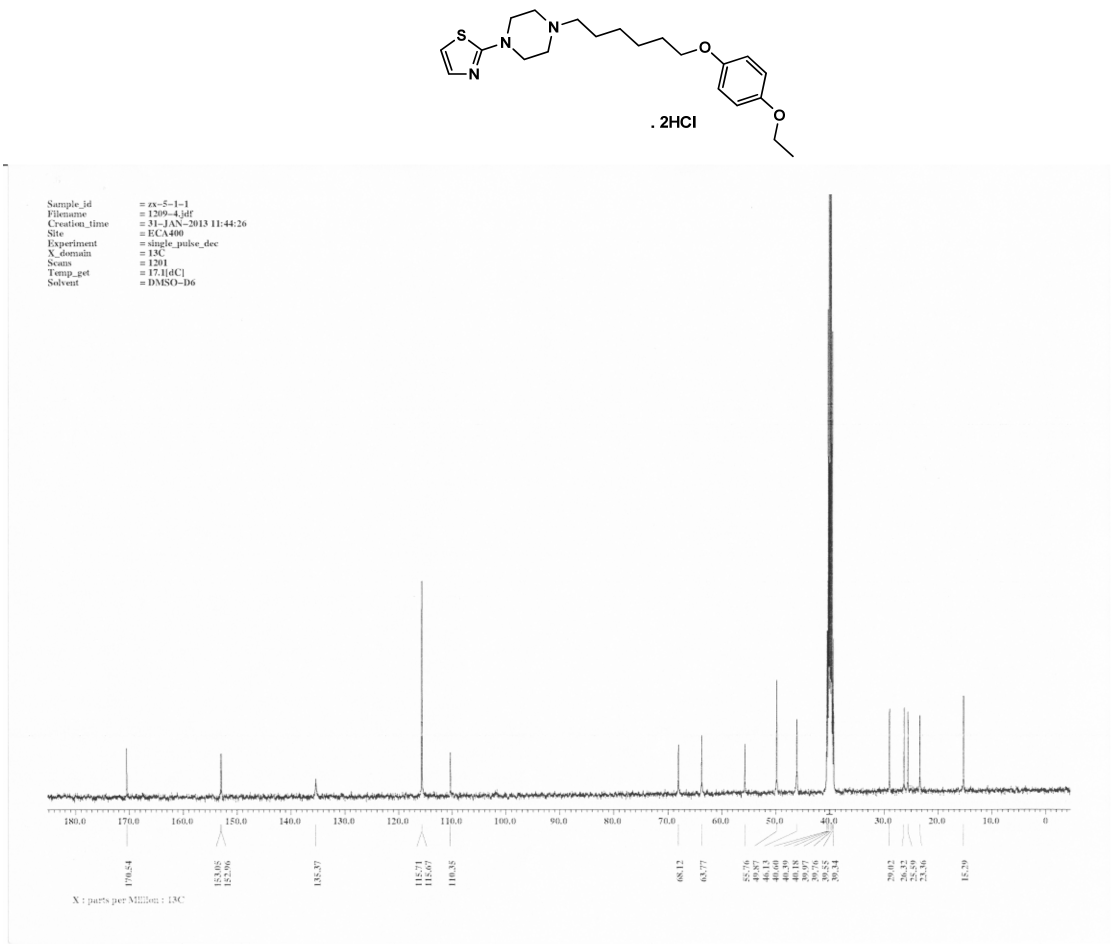

Figure S9. The ESI-HRMS of compound **8c**.

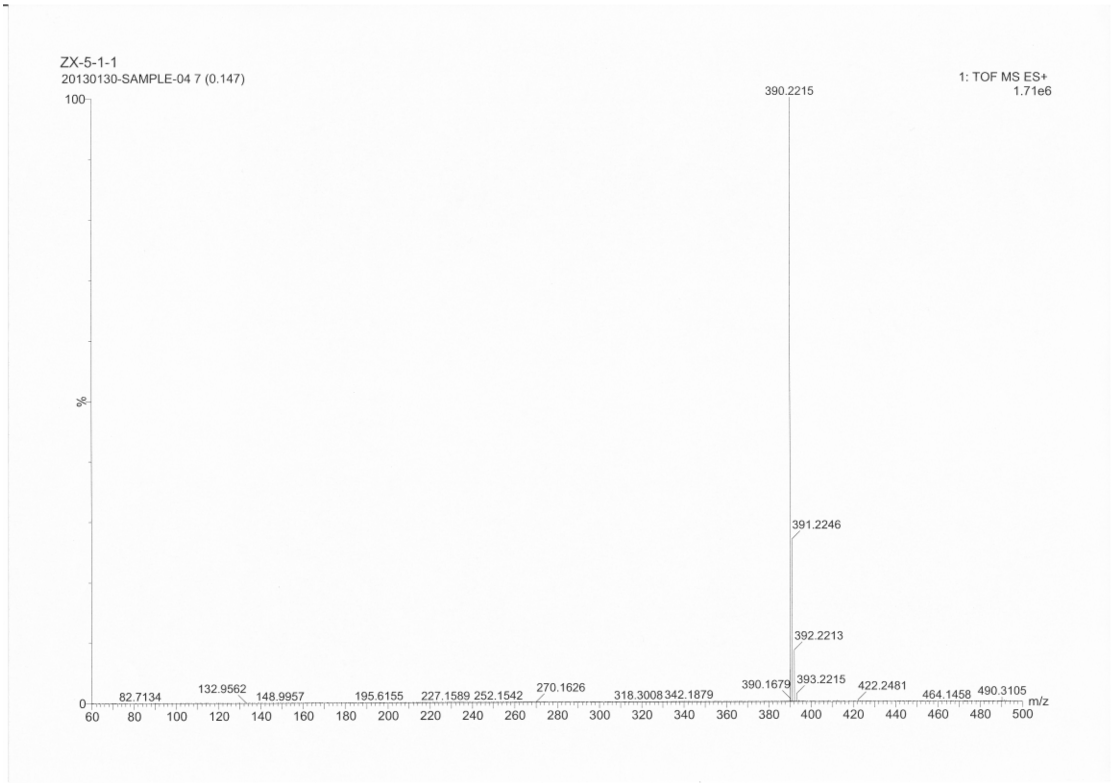

Figure S10. The ^{1}H-NMR spectrum of compound **8d** in DMSO.

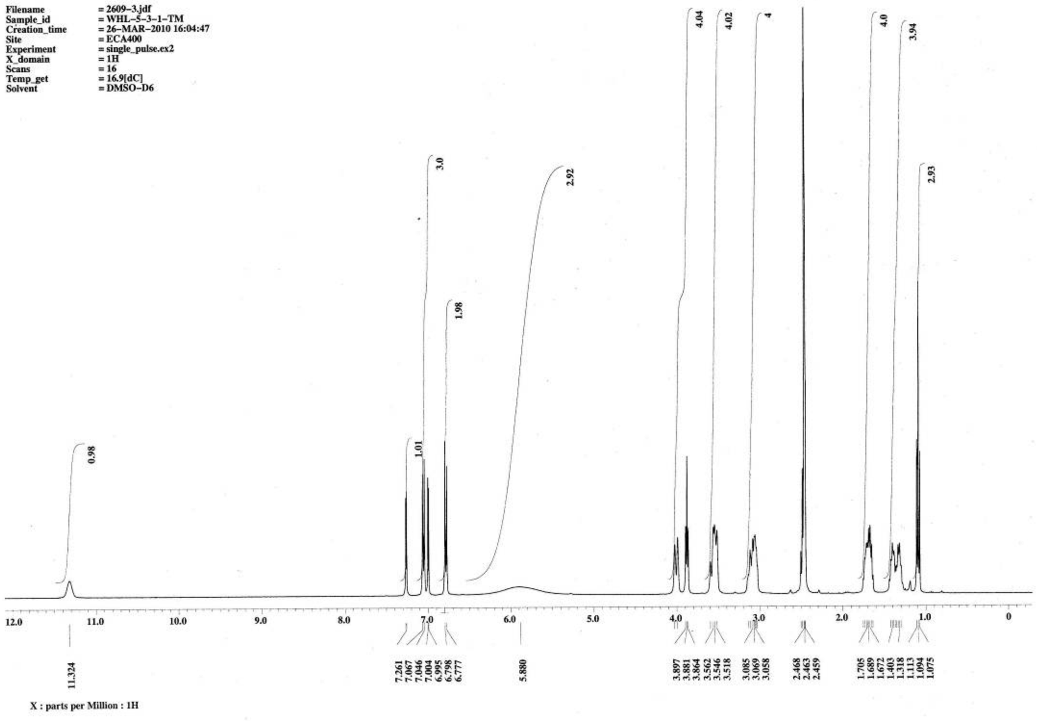

Figure S11. The ^{13}C-NMR spectrum of compound **8d** in DMSO.

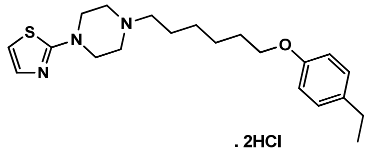

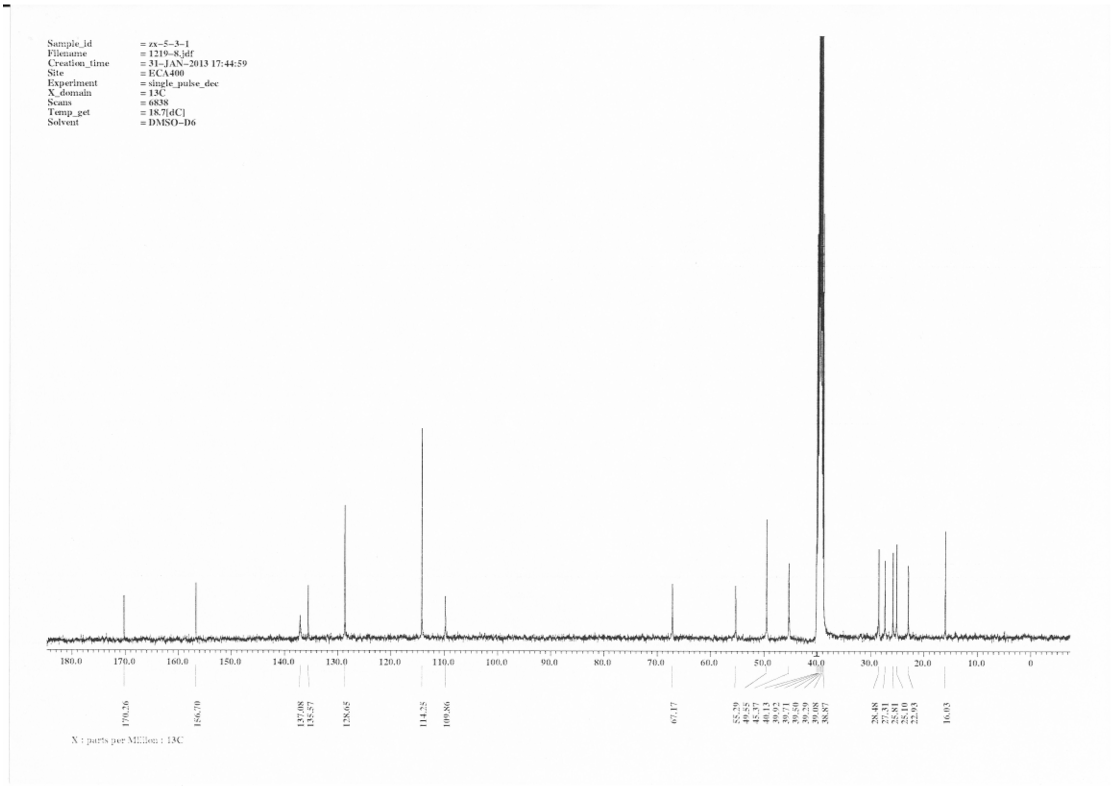

Figure S12. The ESI-HRMS of compound **8d**.

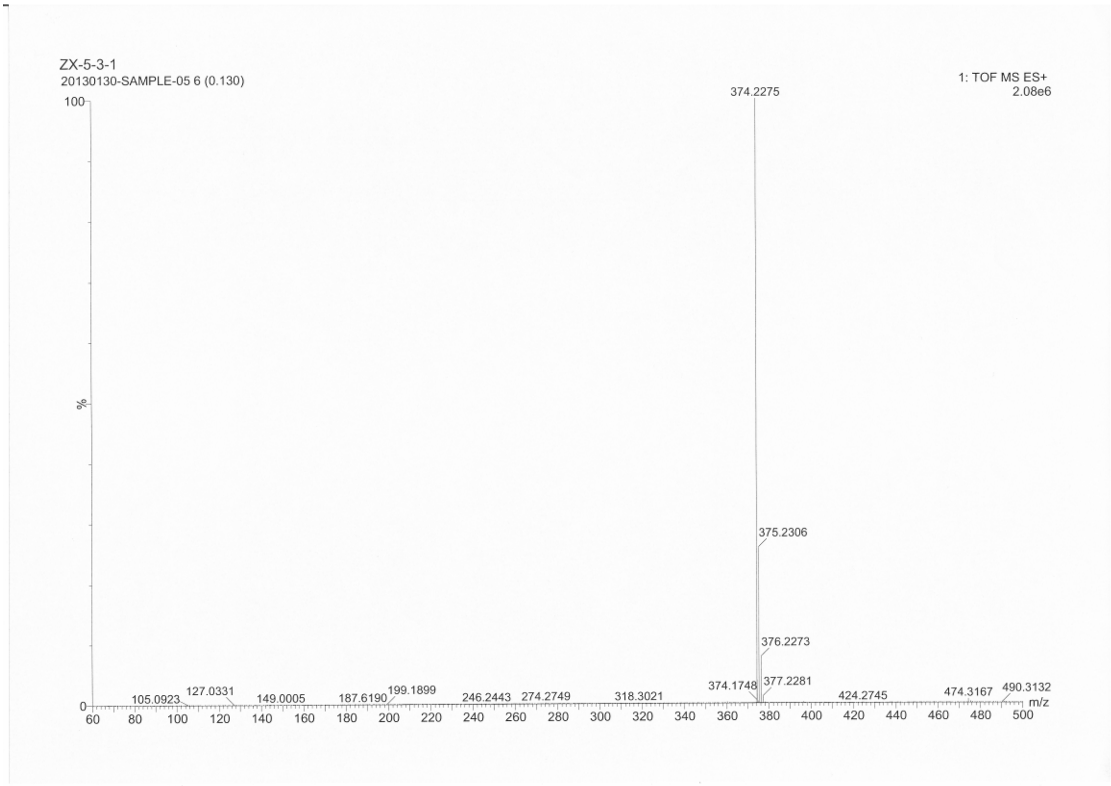

Figure S13. The ^{1}H-NMR spectrum of compound **8e** in DMSO.

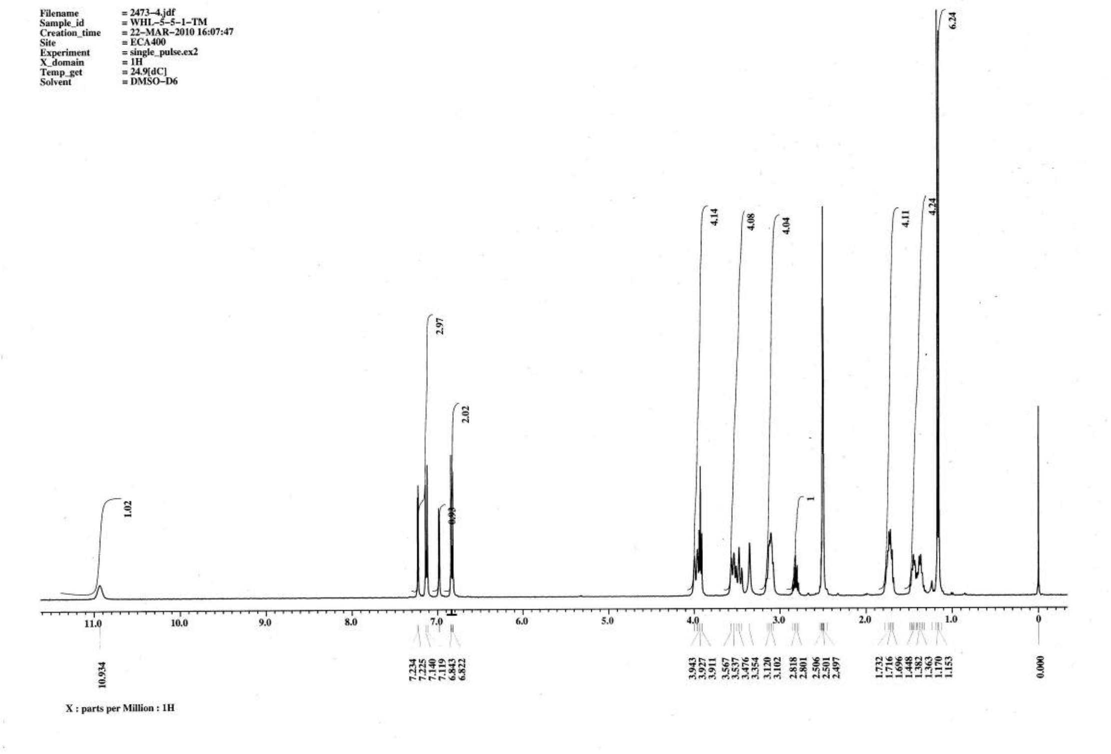

Figure S14. The ^{13}C-NMR spectrum of compound **8e** in DMSO.

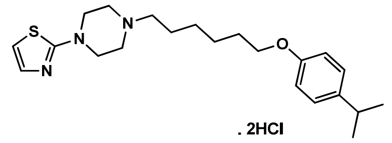

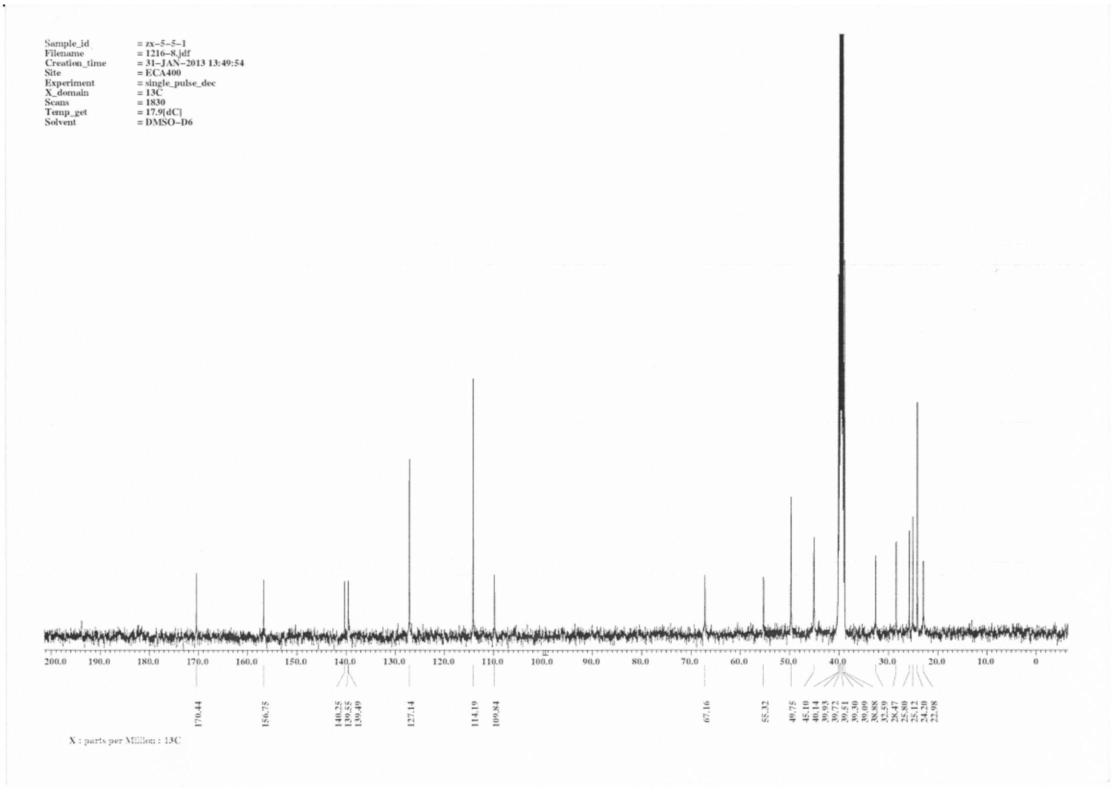

Figure S15. The ESI-HRMS of compound **8e**.

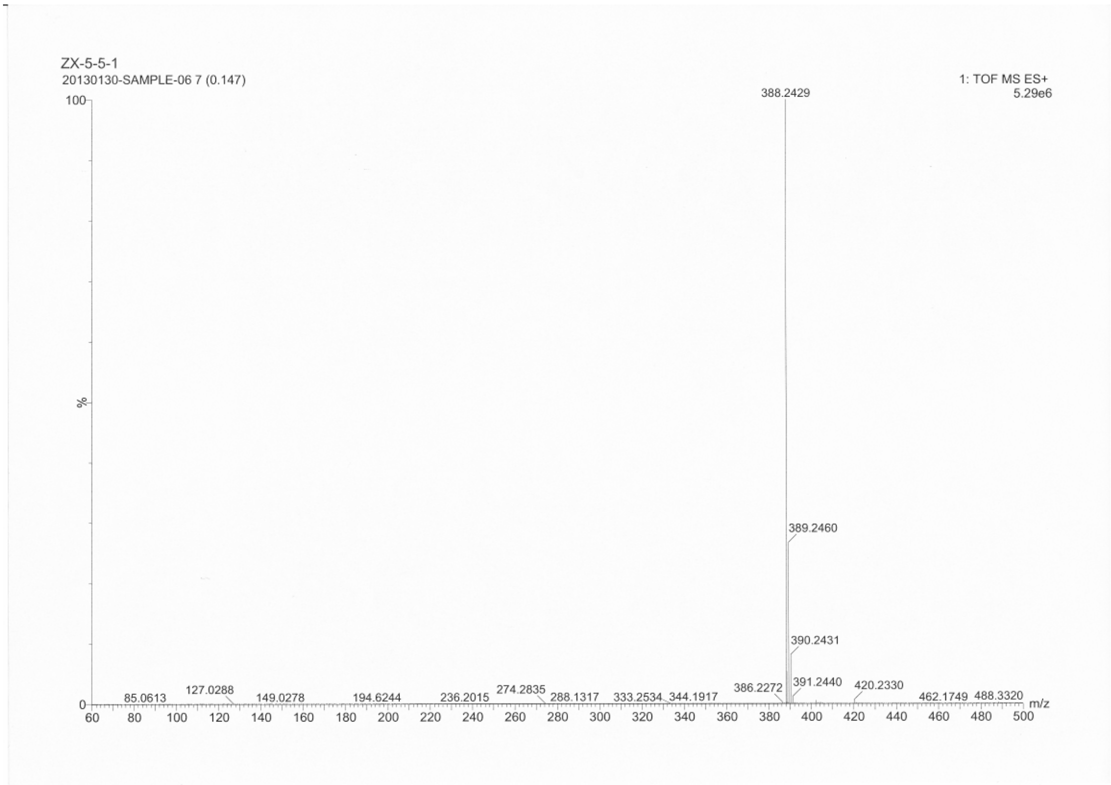

Figure S16. The ^{1}H-NMR spectrum of compound **8f** in $CDCl_3$.

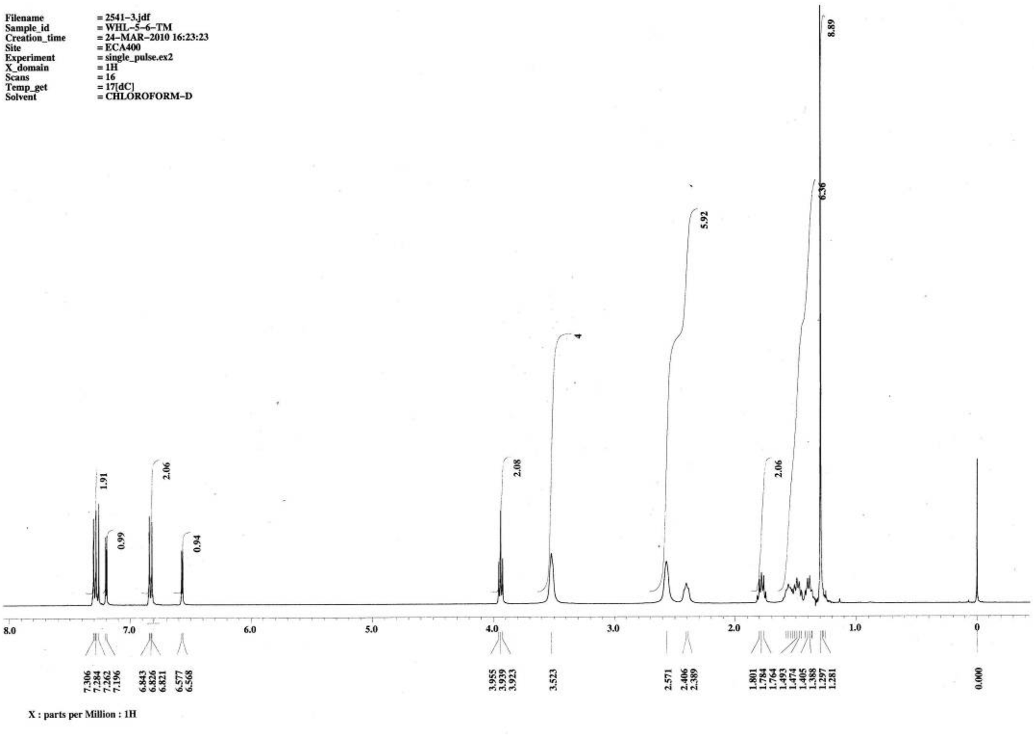

Figure S17. The ^{13}C-NMR spectrum of compound **8f** in DMSO.

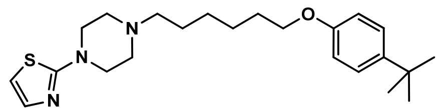

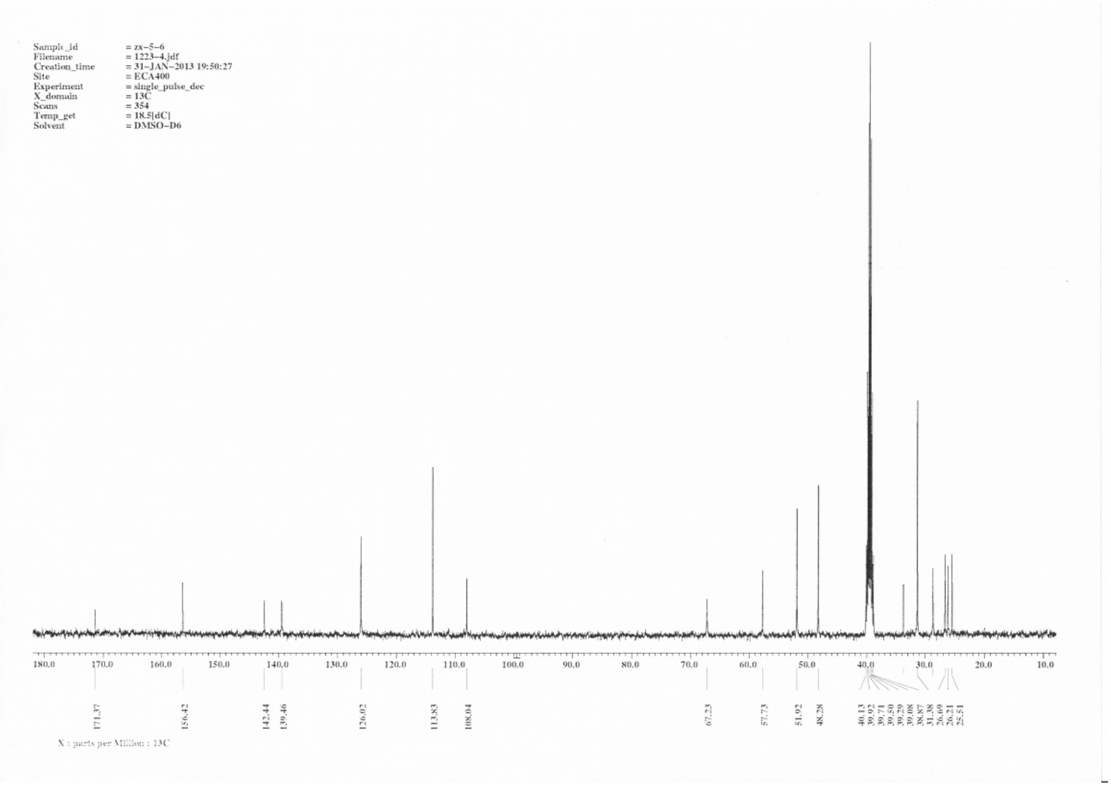

Figure S18. The ESI-HRMS of compound **8f**.

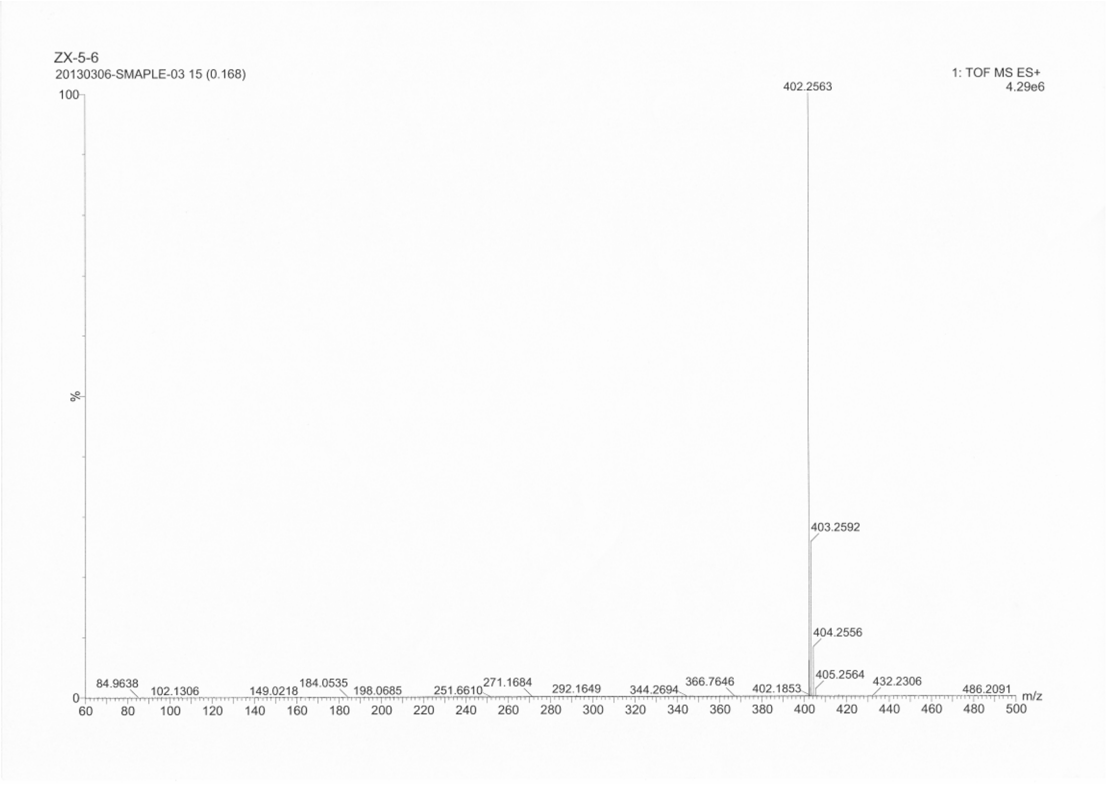

Supplement: Supplementary file 1 [file molecules-18-05059-s001.pdf]
